# *“Either you feel bad and stay*,* or you’re smart and leave” -* perspectives from emergency department staff in Stockholm, Sweden

**DOI:** 10.1186/s12913-026-14871-x

**Published:** 2026-06-03

**Authors:** Clara Brune, Pernilla Lundmark, Lotta Nylén, Martina Gustavsson, Bo Burström, Johan von Schreeb, Ann Liljas

**Affiliations:** Department of Global Public Health, Karolinska Institutet, Tomtebodavägen 18B, Solna, Stockholm, 171 65 Sweden

**Keywords:** Ethical challenges, Emergency care, Psychosocial wellbeing

## Abstract

**Background:**

Job attrition and burnout among healthcare staff is a substantial challenge for healthcare systems globally. Staff working in emergency departments (ED) face work environment challenges including moral distress. This study explores causes, consequences, and contextual dimensions of moral distress among ED staff.

**Methods:**

A total of 36 healthcare professionals from EDs at two hospitals in Stockholm, Sweden, participated in the study. Seven interprofessional focus group sessions were conducted. These were audio-recorded, transcribed verbatim, and analyzed using reflexive thematic analysis.

**Results:**

Participants described providing care under conditions of limited resources and high demands. Drivers of moral distress included inadequate nursing care, futile care, ethical dilemmas, organizational barriers, experiencing team-related challenges and lack of control. Ethical challenges were particularly evident in the care of vulnerable groups, especially geriatric and palliative patients. Participants identified systemic factors such as organizational errors, long waiting times, insufficient staffing, and lack of hospital beds as contributors to moral distress. Reported consequences included worsened well-being, a negatively influenced self-image, delayed reactions, and emotional numbing. Some participants expressed intentions to leave their roles, alongside frustration and distrust toward the healthcare system.

**Conclusions:**

Moral distress emerged as a persistent and normalized feature of ED work, percieved to be primarily driven by resource limitation. These issues need addressing on a policy level. In parallel, strategies to manage moral distress in the ED setting are needed. This has implications for individual healthcare workers’ well-being and organizational stability.

**Clinical trial number:**

Not applicable.

**Supplementary Information:**

The online version contains supplementary material available at 10.1186/s12913-026-14871-x.

## Introduction

Emergency departments (EDs) are healthcare environments marked by several work-related challenges for staff [1, 2]. Previous studies examining the working experiences of healthcare staff in Stockholm’s EDs have identified moral distress, a negative reaction to being unable to act in accordance with one’s values, to pose a pressing issue [3, 4]. Extensive moral distress has been linked to negative outcomes such as depression and burnout [5, 6], and studies point to associations between moral distress and intention to leave the workplace [7]. While moral stress - a normal reaction to morally challenging situations - can be considered an inherent part of healthcare practice [8], moral distress refers to a negative reaction to persistent and unresolved moral stress. Studies suggest that moral distress is particularly prevalent in high-pressure environments such as EDs [2, 9]. In turn, studies indicate that elevated levels of occupational stress, reduced job satisfaction, and increased turnover rates are more prevalent among healthcare professionals working in EDs compared to those in other healthcare settings [10–12]. Additionally, the perceived burden of moral distress can vary depending on organizational, interpersonal, and contextual factors. Some of these factors can be targeted through organizational measures, and understanding causes and contributing factors can be valuable to manage morally challenging situations and prevent negative secondary outcomes [8]. The pressured work environment in Swedish EDs, including the impact of moral distress on staff well-being, has been a recurring topic in national healthcare debates [13, 14], and healthcare workers in Stockholm have signaled an escalating burden put on staff, as well as undermined patient safety [15]. Our previous study exploring moral distress among ED staff during the COVID-19 pandemic found that the issue predated the pandemic. Supporting staff under normal working conditions has been regarded as particularly important given the current and projected global shortfall of healthcare workers, driven by population ageing, retirements, and burnout [16]. There is a need for a more comprehensive understanding of moral distress in the ED setting to provide a foundation for evidence-based and context adapted measures. While prior research has addressed moral distress in other healthcare settings, the specific dynamics of EDs are less understood. Furthermore, most previous studies have focused on individual occupational groups - primarily nurses - and have not adopted an interprofessional approach [17]. This qualitative study explores how ED staff in Stockholm perceive and experience moral distress, with particular focus on its causes. Departing from staff perspectives, this study advances understanding of contextual and systemic factors driving moral distress in Swedish EDs. This increased understanding can inform contextually adapted strategies to support staff well-being and patient care.

## Methods

The study was based on focus group interviews conducted between March and May 2024, at two EDs in Region Stockholm. A total of seven focus groups were held, with 36 participants in total, each focus group consisting of 3–7 participants. Table 1 provides an overview of the participants’ professions and professional experience.

**Table 1:** Study participants, table adapted to not identify participating staff. From left to right: Profession (Nurses (*N* = 12), Doctors (*N* = 16), Assistant nurses (*N* = 8), participant number, years of work experience among participants in each profession

Profession	Participant number	Years of work experience among participants
Assistant nurses (*N* = 8)	7,13,17,25,26,32, 33,36	< 1 (*N* = 3)
		1–2 (*N* = 0)
		3–5 (*N* = 2)
		5–10 (*N* = 1)
		> 10 (*N* = 2)
Doctors (*N* = 16)	2,3,4,6,10,11,12, 19,20,22,23,27,29, 30,31,34	< 1 (*N* = 4)
		1–2 (*N* = 3)
		3–5 (*N* = 3)
		5–10 (*N* = 4)
Nurses (*N* = 12)	1,5,8,9,14,15,16, 18,21,24,28,35	< 1 (*N* = 4)
		1–2 (*N* = 2)
		3–5 (*N* = 2)
		5–10 (*N* = 3)
		> 10 (*N* = 1)

### Recruitment and study population

Inclusion criteria for participation were having a current employment, full-time or part-time, as a physician, nurse, or assistant nurse at either of the emergency departments. All focus groups were arranged to be interprofessional to maintain the interprofessional study approach. Participants were invited via email by the research team, sent to all employees at their respective workplaces. Additionally, CB attended workplace meetings to inform staff about the study. Employees interested in participating contacted CB, who provided potential dates for participation. Once at least three participants were available on the same date, a focus group was scheduled.

### Data collection

Prior to the focus groups, participants watched an eight-minute-long video describing the research project of which this study is a part. As the concept of moral distress is unknown to many, and to ensure a shared understanding of the phenomenon for the discussion, the video described that moral stress is a reaction to in different ways not being able to act in accordance with one’s values [18]. As moral *distress* and -*stress* are synonyms in Swedish (“etisk stress”), no distinction was made in the video between the concepts. The two facilitators, CB and PL, moderated all focus groups. Both are women with a medical background. CB has prior experience of qualitative research and conducting interviews. CB guided the discussions, while PL took field notes. The focus groups were held in meeting rooms at the respective workplaces and lasted on average 83 min. CB started the conversation by asking participants to introduce themselves (name, profession, and work experience) and to share a word they associate with moral distress. Participants were then encouraged to discuss situations involving moral distress. The discussions were guided by the words “*Situation*,* Choices*,* Emotions*,* Causes of moral distress*,* Other Stress*,* Management*,* Consequences*” written on a printed paper placed on the table. These words were chosen to capture the main areas of focus in a semi-structured interview guide developed by the co-authors for the study, based on previous research on moral distress (see supplementary file 1). CB referred to questions in the guide if the discussion had not already covered the topics. All focus groups were audio-recorded and transcribed verbatim by the facilitators. Immediately after each focus group, CB and PL individually documented their reflections and observations in field notes before discussing their impressions. After each focus group, CB and PL discussed potential tentative themes and subthemes with one another, to enable judgement of information power. After five focus group sessions, it was deemed that more data were needed to further reach information power due to the multitude of situations and examples discussed, which resulted in two more sessions.

### Methodological approach

Data were analyzed using reflexive thematic analysis (TA), and reporting of analysis was guided by Reflexive Thematic Analysis Reporting Guidelines (RTARG) [19, 20]. Reflexive TA, developed by Clarke & Braun, is an elaboration of their initial thematic analysis presented in 2006 [21]. The method is flexible and draws on the strength of the researcher’s subjectivity as an incorporated and inevitable part of the analysis process. The method operates within a “Big Q” paradigm, departing from constructivist theories, distancing itself from positivist approaches to knowledge and associated quality assurance. Clarke and Braun emphasize that reflexive TA should be applied creatively rather than procedurally, while maintaining coherent and aligned decision-making across the research process, including the researcher, study design, theoretical foundation, analysis, and reporting [20, 22–24]. Decisions on analysis and study design, including the interprofessional approach and provision of information on moral distress prior to the focus groups, were made based on these principles. In reflexive TA, reflexivity of both the researcher, the process and the context is considered a fundamental part of shaping the research [22]. CB, who mainly conducted the analysis, has previous work experience from one of the involved EDs as a medical student and working as an assistant nurse and junior doctor. This information on CBs professional role was disclosed at the start of the focus groups.

### Data analysis

The analysis followed the six-phase process of reflexive TA [22], primarily adopting an inductive approach. The analysis process included several co-researchers, who were involved in different phases, described in Table 2. Participants’ own descriptions of when they experience moral distress is what guided the analysis, which was deemed congruent with this study’s constructivist epistemological approach. See description of the six phases below (Table 2).

**Table 2:** Study application of the six data analysis phases, as outlined by Clarke and Braun

*Familiarization* with data was conducted by CB to get an overview of data and was done by repeated listening to recordings and reading transcripts, during which additional initial reflexive notes were taken.
*Generating initial codes* was conducted through mainly inductive coding of all data, coding for both manifest and latent content. Coding was guided by the aim of achieving `meaning´ of data*. Initial coding of two transcripts was done first in separate by CB and PL. The researchers then reviewed each other’s codes during physical meetings and had reflexive discussions on how data was interpreted, to enhance reflexive awareness during the following coding-process. The analysis process was then continued by CB who coded transcripts in parallel to writing reflexive notes attached to codes. Codes were then clustered, grouped and rearranged, along with relevant data and reflexive notes. To integrate reflexivity, achieve transparency, crystallize data and enable co-researcher reflections by LN and AL, a comprehensive summary of the data was written, structured by the arranged codes. The comprehensive summary included summarized conversations, quotes and example-notes. *Coding was guided by participants’ reports of moral distress,* meaning that no normative ethical analysis was applied.*
*Identifying themes* was done in parallel to all other phases of the analysis process. According to Clarke and Braun, themes are meaning-based stories while subthemes convey important parts of the story and are used to highlight a dimension of the central concept of the theme (24). While the questions guiding the focus group studies enabled categorisation of data according to subthemes, the `meaning´ reflected in the names of the subthemes was derived from the analytic process. Tentative naming of themes and subthemes were discussed and drafted by the facilitators after each focus group, to enable determination of whether information power had been reached. CB continued to draft overarching themes while coding data. Themes were further elaborated by reviewing the arranged codes and the comprehensive summary. A final review of original transcripts, fieldnotes, reflexive notes and initial codes was also done leading to some refinement of wordings and arrangement, after which preliminary final overarching themes with subthemes were drafted. Themes, subthemes and codes were then arranged into a table (Table 3).
*Reviewing themes* was done in collaboration with co-researchers AL and LN, who have backgrounds in research on work environment and public health. The themes along with the comprehensive summary, and original transcripts were shared with AL and LN who subsequently shared reflections on CBs interpretations. These reflections were considered in the process of *defining themes.*
*Defining themes* was done after receiving further co-researcher reflections by AL and LN on the phrasing of tentative themes, subthemes and codes.
*Producing the report* was done guided by the study purpose, current literature on the topic, final analysis and results, and in collaboration with co-authors who have expertise in the field.

### Ethical considerations

Participants were given information in writing on their rights to withdraw in an information sheet prior to the focus groups. Due to the sensitivity of the topic and the potential need for support after having shared own experiences of moral distress, the information sheet also included information on how to access support. Written or verbally recorded consent was collected from all participants. The study obtained ethical approval through the Swedish Ethical Review Authority, diary number 2023-02555-01.

## Results

The overarching theme along with subthemes and clustered codes are presented in Table 3. Each subtheme is presented below.

**Table 3:** Theme, subthemes and clustered codes generated by the reflexive thematic analysis of data

Theme	Subtheme	Clustered codes
**Experiencing moral distress** - A normalized part of fulfilling the mission	**Causes** - patients suffer when staff are unable to meet the patients’ needs	Prioritization under pressure
		Ethically unaligned decision-making
		Resource availability and allocation
		High workload and a reoccurring gap between demands and capacity
		Lacking teamwork & skills
		Vulnerability & loss of control - extinguishing fires
		Inadequate nursing care
		Feeling insufficient
		Vulnerable patient groups
		An unhelpful visit
		End of life care & treatment limitations
		Organizational barriers
		Extreme events & challenging situations
		Dilemmas and non-compliance with ethical principles
	**Consequences** - a downward spiral	A wish to quit working
		Worsening well-being and influenced self-image
		Delayed reactions and numbing
		Conflicts between work and personal life
	**Context** - the dynamics shaping healthcare	Healthcare in society
		Emergency department specific challenges
		Processing the pandemic
		The patients we work with

### Causes - patients suffer when staff are unable to meet the patients’ needs

Participants described that ED work involves constant prioritization between patients and tasks, and discussed how ED work is characterized by several types of stress. They discussed differences and similarities between stress and moral distress, highlighting that although a high workload could be a driver of moral distress as well as other types of stress, they constitute different experiences. A high workload was reported to increase levels of distress as it involved more frequent and pressured prioritization between patients. Examples included delayed assessment of patients and decreased nursing care despite extensive needs.

...it is hard to experience regular stress without an ethical factor involved as we are always working with people. And whatever you do, you are prioritizing between two people, or patients. - Participant 34.

Participants discussions centered around the difficulties of defining moral distress and distinguishing it from other types of stress and distress. Still, making decisions unaligned with their own values was described as one source of moral distress. These decisions included prioritizing organizational needs - such as preserving resources and hospital beds. Participants also reported experiencing moral distress when having to follow or implement someone else’s decision not in line with their own assessment or values. Doctors reported experiencing such situations with senior colleagues, while nursing staff described experiencing such situations with doctors. Joint decision-making was generally perceived as alleviating, particularly in socially complex situations - such as the discharge of patients to challenging living conditions, due to absence of a clear medical indication for admission.

Participant 4: It can be really nice to be two in that situation, so you sleep a little better.” Participant 2: “Yes, really, to send out people who are homeless, that’s…

Participants reported that a key underlying cause of moral distress was resource scarcity, which negatively influenced several other factors and causes.

Facilitators: …what resources do you perceive are lacking?

Participant 23: Staff. People. … And hospital beds, which is actually due to staffing.

Participant 22: The number of people but also experience.

They reported that patients’ needs were met to varying degrees depending on staffing levels and workload. Participants discussed long waiting hours and lengths of stay in the ED as a key driver of moral distress in the setting. Participants from one of the workplaces referred to a certain week during which there had been sufficient staffing, which had substantially shortened the waiting time and improved the work environment during those days. Participants repeatedly expressed feelings of providing undignified care due to resource scarcity in terms of lack of staff and long waiting time. They also reported experiencing moral distress due to a high workload. The workload was considered high when there were large numbers of patients or when patients had extensive needs.

For me, a typical stressful situation is when you have 40 patients. Well, it doesn’t even have to be 40 patients - it could be having five patients with dementia on the same shift. And you know they are vulnerable, it’s risky for them to be here. They could fall, they could get hurt, they could disappear. And it’s impossible to keep track of everyone the way we have to. - Participant 28.

Participants compared their workload to other healthcare units and discussed the unpredictability, and difficulty to quickly adjust resource availability in response. They reported that under heavy workloads, they frequently applied principles intended for disaster medicine, thereby deprioritizing measures they believed should normally have been prioritized in routine care. They also reported lacking time to guide colleagues. Participants further discussed how suboptimal teamwork with insufficient leadership, lacking communication and disorganized work aggravated the situations. They also discussed how own limitations to capability, such as motivation, personal needs, knowledge, and competence, restricted their ability to provide what they frame as `good care´, and caused moral distress.

It’s not reasonable because it’s not safe, but at the same time, I know that if I don’t do it, nothing will be done. …like should I start providing care that I can’t handle because we are in a resource shortage situation? This isn’t uncommon, I think, especially during nights or holidays, and then you go home, and you have a stomachache thinking, ‘Was that okay or not?’ - Participant 31.

Feeling insufficient and experiencing loss of control in their work was reported, particularly in situations where staff lacked an overview of the severity of their patients’ conditions. Participants further discussed experiencing loss of control when handing over patients to colleagues, discussing trust in colleagues and their assessments. They also experienced loss of control due to lack of information, caused by for example uncoordinated information systems. Participants discussed extreme situations in their work and provided examples of when vulnerability became especially evident.

…and there were way too few people - there was only one person who had to handle a lot of patients at the same time. Running out to the reception, you know, while also performing CPR - at that point, you feel frustrated. You go home with a heartache, and it will last for many, many years. - Participant 13.

Participants further discussed inadequate provision of nursing care in the ED. They gave examples of situations when they did not have time to provide food, change diapers or provide an acceptable social interaction or emotional support to patients. Both nursing staff and doctors described having to prioritize medical needs over nursing care and that focus on person-centered care was lost in the ED work. An inability to manage all tasks expected of them was a recurring topic. They reported that the constant need to prioritize between tasks and patients and being needed at multiple places at the same time, led to feelings of insufficiency.

…With all your ambition and will, you want to make sure they’re not lying there soaked in urine when they’re transferred to geriatrics, but you don’t have time to get to them. Because one patient is septic in the monitoring unit, and you have to prioritize getting antibiotics for them. Or someone falls and gets hurt, and that patient is transported to geriatrics while you’re helping someone else. I’m just trying to say - I have two hands. I can’t be at multiple places at once. But still, you know that the patient hasn’t received what they need, because you were insufficient. - Participant 28.

Participants reported experiencing moral distress when providing care to vulnerable patient groups. These included frail geriatric patients, palliative patients, psychiatric patients, patients with substance abuse or socially vulnerable situations, patients with a presumed difficulty in navigating the healthcare system and patients with language barriers. Staff prejudice and patient language barriers were also discussed to reduce quality of care. Participants described difficulties in using interpreter services, misuse of such or inappropriate translation via family members. Participants further discussed how vulnerability and prejudice influence the care provided to different patient groups.

…I also think of other categories, besides those who clearly need an interpreter - those who are vulnerable, homeless, and struggling with addiction. For me, they end up in the same category, in terms of how we unconsciously handle symptoms, diagnostics, and… well, we’re not as open-minded when considering differential diagnoses. - Participant 23.

Another issue raised was the perception that some patients did not benefit from, or were directly harmed by, their ED visit. Participants discussed moral distress due to providing unnecessary or futile care and reported frustration due to how resources were allocated. A recurring example was patients referred to the ED from geriatric wards or homes, for whom participants perceived pursued care was inappropriate, as well as palliative patients.

… I’m thinking about when we receive Agda, 95, from a nursing home with… It’s like, what are we doing here? How did it come to this - that society… who made the decision to send her here? - Participant 9.

It was further reported that providing end-of-life care was a commonly occurring phenomenon in the ED, and a source of moral distress as it was considered undignified to provide such care in an ED. This was due to patient isolation and perceived lack of competence from ED staff to provide palliative care. Additionally, that the ED visit could lead to unnecessary interventions and futile care.

If it were up to me, I’d want to die in my own bed. I don’t want to die in an emergency department, it’s not dignified. - Participant 7.

Participants also reported perceiving decisions on treatment limitations to be arbitrary due to insufficient information or a lack of patient involvement. This was often caused by time constraints or patients’ cognitive impairment.

…you haven’t seen the patient for that long, but you feel pressure to make certain decisions. - Participant 30.

So, it’s clear that you want to make the right decision. But it’s quite often that there’s a desire to make a decision based on very shaky grounds. - Participant 31.

Participants also reported that family members having opinions on treatment limitations caused moral distress, when these were in conflict with the medical judgement or the patient’s preferences. Further, not having time to provide sufficient support for relatives in extreme situations and not having the time to contact relatives and provide them with adequate information caused moral distress. Participants also discussed organizational obstacles, and how administrative factors directly or indirectly interfered with patient care or needs. Examples included difficulties in finding a hospital bed.

… when patients are bounced back and forth between different specialties. When you know this is a patient who needs to be admitted. It doesn’t matter if it’s surgical, medical, or infectious diseases - they need antibiotics. And yet, everyone keeps passing them around. - Participant 6.

Participants reported needing to work around the system and finding loopholes to provide what they perceived as adequate care. Participants also discussed extreme events in the workplace and reported that they frequently occurred. However, these situations did not necessarily lead to moral distress. For example, events such as cardiac arrests, death of a young patient, or conveying difficult information to relatives did not necessarily cause moral distress if the situation per se could not be influenced. However, experiencing resource shortages, or having to prioritize in conjunction with these events, caused moral distress.

Participant 2: …cardiac arrest in a young person, it made me think. It’s awful when it happens, but maybe it’s not stressful in the same way because you haven’t…” //Participant 6: “No, that’s not moral distress.” // Participant 2: “…personally done anything wrong, it was just that the patient was unlucky, had heart disease and was young, or something.

Participant 6: But then there was someone - someone who died behind the curtain after waiting 12 hours…

Participants further discussed conflicting ethical principles and experiencing moral distress due to failing to act in accordance with them. One example was compromised patient autonomy when performing a medically necessary intervention on a demented patient who resisted.

… it’s not a positive feeling when you leave work feeling like you’ve almost committed an assault on someone who didn’t want it [the intervention]. It goes against your own values. - Participant 24.

Another example was the patient’s right to information, such as when healthcare staff delayed sharing available information for the patient’s benefit. Other dilemmas involved the decision to perform interventions outside one’s competence, if the patient would not receive the intervention otherwise.

### Consequences - a downward spiral

Participants reported that moral distress led to a desire to leave their work. They mentioned highly valued and skilled colleagues who had quit due to not feeling well when working in the ED setting. They discussed that colleagues left because they felt they were not the “right” person for the work, due to inability to cope. One participant had stopped working full-time due to moral distress yet continued working part-time.

I can’t handle the moral distress. I think it’s different (looks at Participant 4) because you’ve always been so good at - I’ve always been very impressed by the distance you keep from it. I don’t have that distance. I enjoyed working here and thought about staying. But I can only handle one shift a month now. I can’t handle the moral distress. - Participant 3.

Some participants also reported that without strategies to cope, they would not be able to continue working.

Either you feel bad and stay, or you’re smart and leave. In the end, it’s not worth getting sick because of it. - Participant 22.

They discussed how current working conditions did not compensate for their moral distress and workload, and that staff were therefore seeking to leave the workplace, even if they enjoyed their work otherwise.

I was told not to apply to the emergency department as a junior for various reasons, but also because of moral distress. Like, `the distress you’ll experience in the emergency department… The salary just doesn’t compensate for it.´ - Participant 13.

Participants expressed a desire for a sustainable work life, and that they would probably choose to work elsewhere in the future to achieve this, despite wanting to continue at the ED.

Yeah, you can question, am I doing my best? Can I do my best? Do I want to work somewhere where I can’t be my best self all the time? - Participant 28.

They reported decreased well-being and affected self-image due to not acting in accordance with their values, and due to the sense of having done a poor job. They reported that repeated experiences caused accumulated feelings, and that worsened well-being decreased their working capacity.

...you think less of yourself. And it’s awful that a workplace can have that effect, I think. - Participant 34.

…but when you have this a bit more chronically, for example several shifts in a row, or we had tough time periods like we did in December-January with huge patient flow, patients waiting 12–14 h… When it’s this long-lasting, you get tired, lose energy, lose the desire to go to work, and if it goes on even longer, it starts to affect your self-esteem. (laughs) Am I not good enough? Am I not working fast enough? Do I have everything it takes for the patient? It starts to affect your confidence and so on, I think. - Participant 29.

While some participants with more experience seemed to have developed functioning coping strategies and approaches to moral distress, other participants reported that their experiences of moral distress worsened over time as they accumulated, and that it therefore with time took less to become overwhelmed. They reported experiencing emotional numbing as a consequence, as it was not possible to continue managing patients’ emotions and suffering, or care about patients in the same way as before.

I also think you become a bit… I’m still very new, but I think you get a bit desensitized. … You don’t really have time to stop and see the patients many times, at least that’s how I feel. - Participant 15.

One participant said they had “turned off” their empathy at work, because it was too tough to feel it and not being able to act in accordance. They discussed that this led to suboptimal interactions with patients. Participants further reported feeling torn between work and private life, having to choose between doing a good job and being sufficient in their personal lives. Some reported experiencing an inability to be emotionally present outside of work. They reported frequently working overtime in an attempt to complete all the tasks expected of them. Participants discussed how the negative impact on their private lives created a vicious cycle of worsened well-being, and difficulties in managing accumulating experiences.

… And then you come to work the next day, and you start from here [low]… I always carry those events with me, at least for a week. It’s like they accumulate… - Participant 21.

… I bring a lot home with me. Almost after every shift, there’s so much that I take home with me. - Participant 30.

### Context - the dynamics shaping healthcare

Participants discussed experiencing moral distress in light of several societal and contextual factors. They shared their views on the healthcare system and what they considered as adequate care, noting that perceptions of what a “need” is, are context- and culture-dependent. They discussed the Swedish cultural context, society, their organization and workplace. The discussions focused on the extent of the ED’s responsibilities, as well as their views on how well the healthcare system and public sector function, sharing varying views and perceptions. Some expressed gratitude toward the well-functioning aspects, while other mainly criticized the dysfunctional elements. They discussed issues in other units and how these affected emergency care, mainly focusing on primary care but also geriatric care. Participants expressed a lack of trust in the capacity of other departments to help patients, which they felt placed additional responsibility on themselves. Discussions also centered around how healthcare lacks adaptation to the growing aging population. Participants expressed that they would not have accepted the current quality of care for themselves.

I admit that I get anxious, I don’t want to grow old. I feel like I want to tattoo ‘0-CPR’ on my forehead with a date, and then clearly talk to my loved ones about what I want if I get a chronic illness because I see that’s where things fail a lot. - Participant 29.

Participants further discussed the production demands they worked under and how the main focus was on these demands, rather than on healthcare staff’s experience of whether they provide good care. Meanwhile, they also expressed gratitude for the available resources in their healthcare system and region, comparing them to hospitals in other regions and countries. A substantial part of discussions further revolved around work strategies in the ED and context specific challenges. Participants discussed the unpredictable workload and tasks, as well as challenges with the premises. Examples were noise, a lack of walls and private rooms during procedures, and interactions with patients in corridors.

Yes, it’s really not ideal. When we have to give bad news in a corridor or in a cubicle. It’s very noisy, we’re talking to consultants about delivering cancer diagnoses, and there are low glass walls so everyone on the other side can hear. - Participant 22.

Participants described how ED work could feel isolated, particularly in decision-making. Participants further discussed challenges with the need to quickly shift between situations and emotional states without time to process them. For instance, handling one patient’s grief and shortly after handling another patient’s frustration. They discussed the strain of receiving patients’ frustration toward dysfunctional elements of the healthcare they represented.

Sometimes, I’m too ashamed of the service. I know when I’ve had leading positions during shifts, and when things are really bad, I don’t want to wear my badge because I feel like I can’t stand by it… so I take off the badge. (laughs) I can’t stand for the mess. - Participant 2.

Participants also shared their experiences of moral distress during the COVID-19 pandemic. They compared the current work situation to the altered conditions and the frequent witnessing of death during the pandemic.

Participant 27: Everything feels easy in comparison, maybe.

Participant 28: What’s strange is that we moved on so quickly after that. People still manage to keep working in healthcare after that experience.

They reported that the pandemic had sparked some discussions about moral distress in healthcare, as well as prioritization and treatment limitations. They also discussed positive aspects of the pandemic, such as the professionals themselves making decisions at the workplace and solving problems, in contrast to the usual top-down management.

And for the first time, we as professionals… we handled everything. Management was completely helpless… The professionals took care of everything. - Participant 10.

Participants further discussed their views on patients, including the patients’ responsibilities when seeking emergency care. They reported experiencing that the ED functioned as an assembly point for individuals with both medical and social needs, and acted as an end-station for individuals whose actual needs should be covered by other healthcare units and societal agencies.

A lot of it comes down to societal factors, like, we’re forced to make difficult decisions due to lack of resources. There are no ICU beds, no beds in the hospital, geriatrics are full, elderly care is insufficient, primary care is overwhelmed, and no one trusts primary care due to past poor experiences, and the ED can manage some patients, but not all. And so patients are shuffled around in this mess. The patients are the ones who suffer. Yeah, no one wants to be in the ED. They come here, and I understand why they’re a little demanding. They’ve been sent around in the system the entire day. I saw a patient who had been sent from 1177 [digital service for medical advise], to their GP, to the ED, back to their GP, and then to the ED again - all within a single day. - Participant 28.

They acknowledged that providing emergency care involves filtering out certain needs, which also caused moral distress, due to an inability to help. Participants further expressed both sympathy and frustration towards patients. A recurring example was patients inquiring about waiting times and their position in the queue. They also reflected on their approach to prioritizing patients’ needs, and the dilemma of either providing better care for one patient, or less care to several patients. They considered the long-term impact of their actions on both the individual patient and society, where time spent on a single patient could represent a long-term investment in that patient’s health with positive consequences for the healthcare system and society, but acknowledged that the ED mission did not allow for such long-term perspectives.

## Discussion

This interprofessional focus group study gathered perspectives from ED staff regarding moral distress. Participants shared a range of situations during which they experienced moral distress due to providing insufficient care, with resource constraints frequently cited as the underlying cause. Management of geriatric patients and provision of end-of-life care were recurring topics, with participants expressing that they regularly faced situations they perceived compromised patient dignity. These experiences were reported to negatively influence personal well-being and motivation to continue working in the ED. Overall, participants shared the view that experiencing moral distress was an inherent part of working in healthcare, which with time became normalized. Focus of discussions was directed toward system-level drivers: while some tragic events were accepted as part of healthcare, systemic deficiencies were criticized for preventing care aligned with patient and staff expectations. The finding of resource scarcity as a root cause of moral distress has been documented in previous studies conducted in EDs in other high-resource settings as well [25, 26]. Workload appears to be a major stressor [1] with bed shortages and limited capacity contributing to job dissatisfaction among ED staff [27]. Additional root causes have been identified in lacking medical knowledge among the public, overuse of emergency services, and shortcomings of the welfare system, all of which leading to increased pressure on EDs and contributing to moral distress among staff [28].

Previous studies have mainly taken on survey- or interview formats and focused on specific occupations. The focus group format of this study allowed for visualized group dynamics, revealing shared perceptions but also differences in opinions across occupational groups. Both nursing staff and doctors reported experiencing moral distress due to acting on others’ decisions within the hierarchical structure, although doctors experienced moral distress due to acting on senior colleagues’ decisions while nurses experienced moral distress due to acting on doctors’ decisions. The interprofessional format further facilitated deeper understanding of these similarities and differences. This is considered a strength of the study due to the broadened perspectives, although it can be assumed that the interprofessional outline inevitably introduced hierarchical power structures. The study outline was guided by the quality markers of *worthy topics*,* rich rigor*,* sincerity* and *credibility*. Sincerity was addressed through self-reflexivity - an approach emphasizing honesty, authenticity and transparency [29]. Reflexive thematic analysis was chosen to acknowledge how CB’s background as a healthcare professional influenced the analytic process. This insider perspective was considered valuable in deepening the contextual understanding, enabling interpretative depth. Credibility, referring to the trustworthiness and plausibility of findings, was supported through thick descriptions, triangulation, and multivocality [29]. Decisions such as representation of varied participant perspectives and involving multiple co-authors in the analysis contributed to this aim. Limitations to the range of perspectives from which the findings can be discussed include the positionality of CB. While this may be viewed as a limitation from a positivist stance emphasizing objectivity, it is not considered a flaw within reflexive thematic analysis, provided that relevant reflexive information is disclosed [22]. Another limitation is the lack of analysis of how power dynamics between groups of healthcare staff may have shaped the focus group interactions - an important aspect that falls outside the scope of this study. Still, an important consideration when interpreting these results is whether some perspectives or voices were not expressed during the focus groups due to hierarchical dynamics.

Although reports of resource scarcity as a key cause of moral distress in this study is consistent with findings from other high-resource healthcare systems [25, 28], context-specific factors likely shaped these perceptions in the present setting. Researchers highlight that recent real-term budget cuts have resulted in declining resource provision across the whole public sector in Sweden [30]. They link this trend to a political and economic paradigm that discourages welfare investment, thereby limiting reinvestment and hindering adaptation to a growing population with increasingly complex healthcare needs [30]. At the same time, the healthcare system is growing in complexity and size, with growth accompanied by a shift in resources toward organizational structures. Although total resource allocation has increased - mainly through higher staffing levels, rising costs and a higher health expenditure per capita - greater proportions are directed toward central administration. This has coincided with reduced direct patient contact, longer waiting times, and lower productivity [31, 32]. Moreover, existing financing and measurement systems primarily emphasize productivity and often fail to capture care quality, making it difficult to promote high-quality care amid ongoing efficiency demands [31]. These processes of gradual decrease in resource allocation, proportionate to the norm of patient needs, are likely to at some point affect patient safety, as reflected in participants’ reports of compromised care.

Participants’ reports align with assessments from the Swedish Health and Social Care Inspectorate (IVO), which has concluded that patient safety cannot be guaranteed at several Swedish emergency hospitals. Inspections reveal widespread issues, including waits exceeding 24 h, overcrowding, and persistent capacity shortages. Additional problems include delayed or missed medication, insufficient monitoring, and unmet basic needs such as hygiene, nutrition, and hydration [33]. IVO links these systemic shortcomings to a chronic lack of hospital beds, a well-documented issue within both general and intensive care units in Sweden [34]. The number of hospital beds has decreased in recent years, and Sweden has the lowest hospital bed rate in the EU [35–38]. This shortage is frequently cited by healthcare professionals as a daily barrier to safe and effective care [39, 40], and was a recurring concern in the present study. IVO further highlights that the persistently high hospital bed occupancy rates pose a serious risk to patient safety, and that while healthcare staff and services make considerable efforts to manage these risks, the underlying problems extend beyond their control [33].

Participants’ discussions were characterized by feelings of frustration and powerlessness, directing focus toward system-level causes of moral distress. This is well aligned with Epstein and Hurst’s framework, which conceptualizes moral distress as a personal experience that reflects system-level problems: *“an alarm bell that some aspect of the situation has gone awry*,* not that staff are under-resilient”.* Epstein and Hurst’s framework has three background conditions for moral distress: *(i) lack of voice*,* (ii) external causes*,* and (iii) moral hazard* - as those making decisions are not the ones bearing their consequences. While Epstein and Hurst argue that healthcare staff acknowledge and accept that resources are limited in healthcare and prioritization is necessary, they also assert that professionals can see when the system unnecessarily fails both themselves and their patients, and advocate for *structural accountability* for such system failure. Based on the study findings, this structural accountability is mainly needed to address the gap between resources that are available and perceived as necessary. Some studies that similarly have found a lack of resources as a main cause, frame this as a gap between demand and capacity [26, 28] while others frame it as a mismatch in expectations - between professional and patient ideals and the care that can be delivered [26]. Regardless, the systematic mismatch reported in this study raises ethical and political questions about prioritization in healthcare. These issues require policy-level attention and consideration of the ethical principles that guide healthcare provision. According to the Swedish Health and Medical Services Act (SFS 2017:30), care must be accessible, uphold high standards of hygiene and quality, and be delivered with respect for patients’ dignity and autonomy. Patients’ needs are to be the primary consideration in clinical decisions [41]. When prioritization is necessary, it should follow the principles of human dignity, need and solidarity, and cost-effectiveness, as outlined in Government Bill 1996/97:60 [42].

Despite this legal and ethical framework, participants described situations in which they felt unable to deliver sufficient care - based on their professional values, their training in what constitutes good care, and the expressed needs of patients. Considering the legal framework guiding decision-making is enacted by the Swedish Parliament as representative of the people of Sweden, questions of care quality and prioritization are inherently political and democratic. Yet, as healthcare needs grow and resources fail to keep pace, the burden of increasingly difficult decisions is shifted to individual healthcare professionals, placing a heavy ethical responsibility on them - causing them moral distress. Another policy-relevant finding concerns organizational shortcomings in how the ED is used, particularly the frequent and, according to participants, often unnecessary referral of palliative patients. These situations were described as causing avoidable suffering, harm, and sometimes futile care. The causes of unnecessary referrals may be attributed to IVO’s findings of widespread deficiencies in eldercare, including weak compliance with end-of-life care regulations and a lack of individualized medical assessments [43]. Since EDs serve as the entry point to hospital care, such shortcomings are acutely visible to ED staff. Their observations provide critical insights into systemic failures in the care of vulnerable patient groups and warrant further investigation.

Participants reported that the emotional impact of their experiences extended beyond work, with considerable time and energy spent processing distress outside of working hours. Some reported that repeated experiences led to a vicious cycle with less resilience to manage continuing moral challenges, in line with theories on *`the crescendo effect´*, a gradually heightened sensitivity to moral distress called moral residue [44]. Some described reduced engagement with patients, and others expressed a desire to leave their jobs, reinforcing established links between moral distress, compassion fatigue, and turnover intentions [6]. This cycle is a pressing issue in the ED setting: a systematic review found that over half of emergency nurses reported having left or considered leaving their jobs due to moral distress [45]. These indications are further worrying considering an expected shortage doctors, nurses, and midwives due to a combination of an aging population - both among patients and healthcare workers - and challenging working conditions contributing to staff burnout and retention issues [46]. At the same time, growing healthcare needs are likely to further intensify staff workload and prioritization pressures. Considering the systemic nature of these drivers, the burden of moral distress is perhaps unlikely to disappear. The study findings in light of these upcoming challenges therefore underscore the need for research into tailored interventions to address moral distress in emergency care, and to examine its long-term effects on staff mental health and retention.

## Conclusion

The findings highlight causes and consequences of moral distress among ED staff in Stockholm, adding to the limited understanding of context-specific and systemic drivers. Causes varied but connected in different ways to access to resources - mainly in terms of staffing, waiting time and hospital beds - as well as organizational errors. Reported consequences of moral distress were worsened well-being with an influenced self-image, and decreased motivation to continue their jobs. These findings are alarming considering the necessity of a robust healthcare workforce. Due to the systemic nature of the factors driving moral distress, these issues require attention at the policy level, while contextually adapted support structures need to be implemented simultaneously. The knowledge generated in this study can inform targeted measures to support staff well-being and improve patient care, as well as reforms across the care chain to reduce pressures on EDs. Examples include strengthened geriatric emergency services, improved coordination between primary care, municipal care, and hospitals, and expanded alternatives to ED visits for residents in long-term care facilities as well as increasing hospital bed capacity and addressing bottlenecks in inpatient flow.

## Supplementary Information

Below is the link to the electronic supplementary material.

Supplementary Material 1

## Data Availability

The data, including interview transcripts, are not available for sharing in accordance with the information provided to participants, which stated that the data would only be accessed by the researchers directly involved in the study.
