# Supplementary material for: “Either you feel bad and stay, or you’re smart and leave” - perspectives from emergency department staff in Stockholm, Sweden
Source: BMC Health Serv Res. 2026 Jun 3;26:793. doi: 10.1186/s12913-026-14871-x (PMC13242116; doi:10.1186/s12913-026-14871-x)
Supplement: Supplementary file 1 — Supplementary Material 1 [file 12913_2026_14871_MOESM1_ESM.docx]

**Appendix 1**

**Questionnaire**

#### **Part 1 of the Focus Group**

1. **Introduction**: Each participant takes a turn sharing a word they associate with ethical stress and explaining why.
2. **Defining Ethical Stress**: How would you describe moral distress?
3. **Personal Experiences**:
   1. Participants, one at a time, briefly describe a situation where they experienced ethical stress while working in the emergency department.
   2. Following this, the group discusses the situation collectively, addressing:
      1. The underlying causes of the stress.
      2. Any available choices or alternatives.
      3. What specifically felt morally distressing in the situation.
      4. Other emotions experienced during the situation.
      5. The presence of other types of stress.
      6. How the situation was managed.
      7. The consequences of the situation.
   3. A sheet with the following headings is placed on the table to guide the discussion: *Situation, Choices, Emotions, Causes of moral distress, Other Stress, Management, Consequences.*

Questions below are posed if they are not already addressed spontaneously:

1. In what situation did moral distress arise?
2. What were the underlying causes of the situation?
3. What choices were available in the situation?
4. What specifically made the situation morally distressing? Were there any factors that eased or worsened the experience?
5. Did you experience other forms of stress? If so, in what way? What caused the other stress rather than moral distress?
6. How was the situation managed? Could it have been handled differently?
7. What were the short- and long-term consequences of the situation? How did it affect your work performance?
8. How did you cope with the moral distress and any additional stress? What do you think is the best way to manage such situations?
9. What are the differences and similarities between moral distress and other forms of stress? Do they influence each other?

*pause, if requested*

#### **Part 2 of the Focus Group**

1. **Current Tools and Practices**:
   1. What tools, priorities, models, or methods currently exist at your workplace to manage morally distressing situations?
   2. What do you think is needed more or less of?
   3. What works well, and what does not?
2. **Prioritization After Ethical Stress**:
   1. What priorities do you consider most important in response to a morally distressing situation?
   2. How should these be addressed in the short term (immediately and during the shift) and long term (after the shift)?
3. **Organizational Changes**:
   1. Do you believe any aspects of the organizational structure (e.g., leadership, care practices, emergency department operations, or hospital policies) should be changed to reduce the risk of moral distress?
   2. What can the organization do before, during, and after a morally distressing situation occurs in the emergency department?
4. **Recommendations for Improvement**:
   1. Based on your current experiences, do you have recommendations or suggestions for how individuals can better manage these challenging situations in the emergency department?
